# Novel Functionalized Cellulose Microspheres for Efficient Separation of Lithium Ion and Its Isotopes: Synthesis and Adsorption Performance

**DOI:** 10.3390/molecules24152762

**Published:** 2019-07-30

**Authors:** Ichen Chen, Chenxi Xu, Jing Peng, Dong Han, Siqi Liu, Maolin Zhai

**Affiliations:** Beijing National Laboratory for Molecular Sciences, Radiochemistry and Radiation Chemistry Key Laboratory of Fundamental Science, Key Laboratory of Polymer Chemistry and Physics of the Ministry of Education, College of Chemistry and Molecular Engineering, Peking University, Beijing 100871, China

**Keywords:** cellulose microspheres, crown ether, radiation grafting, adsorption, separation of lithium isotopes

## Abstract

The adsorption of lithium ions(Li^+^) and the separation of lithium isotopes have attracted interests due to their important role in energy storage and nuclear energy, respectively. However, it is still challenging to separate the Li^+^ and its isotopes with high efficiency and selectivity. A novel cellulose-based microsphere containing crown ethers groups (named as MCM-*g*-AB15C5) was successfully synthesized by pre-irradiation-induced emulsion grafting of glycidyl methacrylate (GMA) and followed by the chemical reaction between the epoxy group of grafted polymer and 4′-aminobenzo-15-crown-5 (AB15C5). By using MCM-*g*-AB15C5 as adsorbent, the effects of solvent, metal ions, and adsorption temperature on the adsorption uptake of Li^+^ and separation factor of ^6^Li/^7^Li were investigated in detail. Solvent with low polarity, high adsorption temperature in acetonitrile could improve the uptake of Li^+^ and separation factor of lithium isotopes. The MCM-*g*-AB15C5 exhibited the strongest adsorption affinity to Li^+^ with a separation factor of 1.022 ± 0.002 for ^6^Li/^7^Li in acetonitrile. The adsorption isotherms in acetonitrile is fitted well with the Langmuir model with an ultrahigh adsorption capacity up to 12.9 mg·g^−1^, indicating the unexpected complexation ratio of 1:2 between MCM-*g*-AB15C5 and Li^+^. The thermodynamics study confirmed the adsorption process is the endothermic, spontaneous, and chemisorption adsorption. As-prepared novel cellulose-based adsorbents are promising materials for the efficient and selective separation of Li^+^ and its isotopes.

## 1. Introduction

Lithium has been used widely as an important component in energy storage devices [1] due to its low atomic weight, high energy density, and high reactivity. Lithium isotopes (^6^Li and ^7^Li with a relative abundance of 7.5% and 92.5%, respectively [2]) play an important role in the development of nuclear energy, ^7^LiOH has been used as a pH controller and coolant in pressurized water reactors, and ^6^Li is a critical tritium breeding material in a fusion reactor [3]. With the increase of requirement in the clean energy power, it is necessary to develop efficient separation methods to obtain plenty of lithium and its isotopes with high purity. In the past decades, the main method to separate lithium isotopes is the liquid-liquid extraction by using crown ether with different cavity sizes as extractant due to its complexing ability towards Li^+^ [4]. Particularly, benzo-15-crown-5 (B15C5) as extractant exhibited good selectivity for the separation of lithium isotopes with a maximum single stage separation factor of 1.044 ± 0.003 [5]. Although, the crown ethers have been proven to be effective extractants in liquid-liquid extraction of lithium isotopes, there are still some disadvantages, such as a large amount of organic solvent used in separation, environmental pollution and loss of crown ether during the extraction. Therefore, the immobilization of crown ether onto different supports has attracted interests in recent years, and become a promising method to separate the lithium and its isotopes [6,7,8,9]. By using chemical functionalization, the oxidized carbon nanotubes were modified with hydroxy-dibenzo-14-crown-4 ether and the resultant adsorbents had the adsorption capacity of 2.11 mg·g^−1^ [10]. Recently, a mesoporous silica modified with the 4′-aminobenzo-15-crown-5 (AB15C5) was produced by chemical modification, which had an adsorption capacity of 5.4 mg·g^−1^ and a separation factor of 1.049 ± 0.002 [11], and they found that AB15C5 ligand has better complexing ability with Li^+^ and good selectivity for the lithium isotopes than those of B15C5. Subsequently, they prepared a novel AB15C5 grafted mesoporous silica via atomic transfer radical polymerization. By controlling the chain length of grafting polymer, the adsorbents showed a maximum adsorption capacity up to 6.97 mg·g^−1^ and a separation factor of 1.038 ± 0.002 of ^6^Li/^7^Li [12], indicating that with the increase of adsorption capacity of Li^+^, the separation factor of lithium isotopes decreased. Besides the inorganic support, various synthetic polymers have been used as a matrix. The polysulfone adsorbents modified with AB15C5 groups was prepared to give a maximum single-stage separation factor of 1.031 ± 0.002 [13]. Zeng et al. incorporated chitosan together with B15C5 into polyvinyl alcohol porous blend membrane, which showed a maximum single-stage separation factor of 1.046 [14]. 

Compared with the synthetic polymers, cellulose is a natural nontoxic and biodegradable polymer with low cost, which is being widely used in a number of applications, especially for applications as adsorbent [15,16,17,18,19]. Cellulose microspheres, which can be used easily in adsorption and chromatographic separation, have enormous potential of candidate applications in the industry [20]. But the molecular structure of cellulose limits its usage in selective adsorption without proper modifications [21]. The synthesis of functionalized cellulosic adsorbents by grafting modification to introduce specific functionalities is one of the most efficient ways to improve the physical and chemical properties of cellulose [21]. Compared with the chemical-initiated grafting methodradiation-induced emulsion grafting as an environmentally, easily controllable and efficient method, without using any initiators and catalyst, is a very promising grafting method to combine various polymers with many substrates [22,23,24]. 

**Figure 1:**
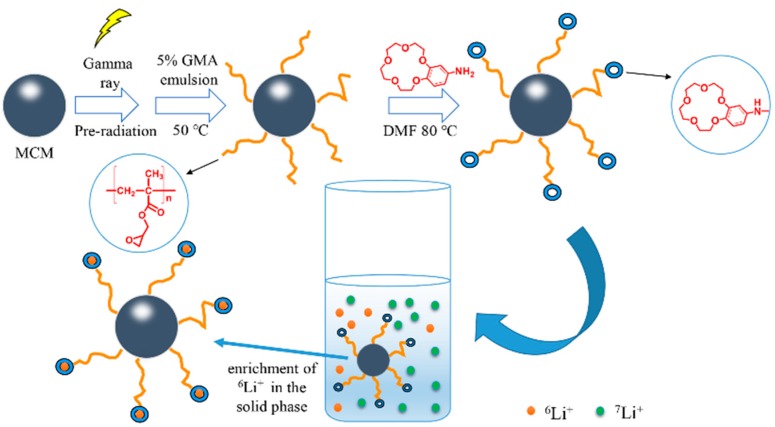
Illustrative synthesis route of MCM-*g*-AB15C5and expected adsorption process towards lithium isotopes.

In the present work, the novel functionalized cellulose microspheres with AB15C5 were fabricated by γ-ray pre-irradiation-induced emulsion grafting and followed by chemical functionalization, with GMA as grafting monomer and AB15C5 as a ligand of Li^+^ and its isotopes. As shown in Figure 1, after pre-irradiation-induced emulsion grafting of GMA, the grafted cellulose microspheres (MCM-*g*-PGMA) were reacted with AB15C5 in DMF by a ring-opening reaction between the epoxy group of grafted microspheres and an amino group of AB15C5 to obtain AB15C5 functionalized cellulose microsphere (named as MCM-*g*-AB15C5). Some parameters that affect the adsorption uptake of Li^+^ and separation of lithium isotopes were studied such as solvent, metal ions and adsorption temperature, and the adsorption mechanism of the Li^+^ was discussed as well.

## 2. Results and Discussion

### 2.1. Synthesis and Characterization of the Adsorbent

**Figure 2:**
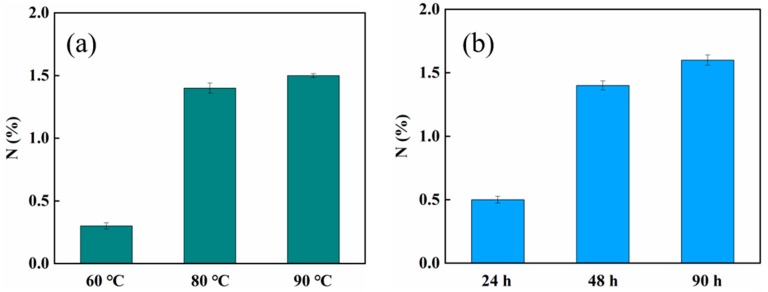
Content of nitrogen element in MCM-*g*-AB15C5 at different reaction temperatures (**a**) and reaction times (**b**).

The synthesis route of MCM-*g*-AB15C5 is illustrated in Figure 1. According to our previous work, MCM-*g*-PGMA with different grafting yields could be prepared by pre-irradiation-induced emulsion graft polymerization of GMA onto MCM by regulating the absorbed dose and concentration of monomer [25]. Considering the content of functional groups and physical performance of functionalized MCM, the MCM-*g*-PGMA with a grafting yield of 81% was synthesized from 2.0 g MCM reacted with 2.1 g GMA and used in the following chemical reaction with AB15C5. In order to favor the nucleophilic substitution reaction between PGMA grafted onto MCM and NH_2_-group in AB15C5, DMF was used as a solvent to improve the ring-opening reaction of PGMA. Then we studied the effect of reaction temperature and time on the content of AB15C5 onto grafted MCM. After the chemical reaction, the N element which only comes from AB15C5 could be measured in the resultant modified MCM-*g*-PGMA, indicating the successful synthesis of MCM-*g*-AB15C5. As shown in Figure 2, the amount of N element in MCM-*g*-AB15C5 increased with the increase of reaction temperature from 60 to 90 °C (Figure 2a) and the increase of reaction time from 24 h to 90 h (Figure 2b). According to the above results, suitable reaction condition is chosen to be 80 °C and 48 h.

**Figure 3:**
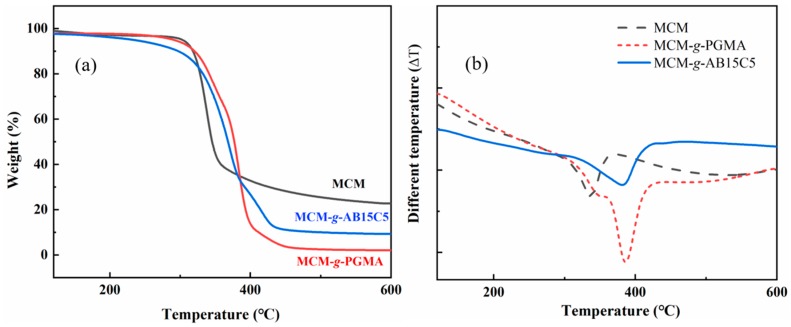
TGA curves (**a**) and DTA curves (**b**) of MCM, MCM-*g*-PGMA and MCM-*g*-AB15C5.

TGA is an important means of characterizing the thermal stability of materials. The TGA curves for the MCM, MCM-*g*-PGMA and MCM-*g*-AB15C5 are shown in Figure 3a. All samples have a mass loss before 100 °C, which is due to the loss of water in the samples. For the studied samples, the decomposition of cellulose happens in the range from 270 to 360 °C [26], and DTA curve of MCM shows an endothermic peak due to dehydration and depolymerization of cellulose (Figure 3b). The epoxy groups and ester in PGMA degraded from 220 to 300 °C [27] and they are corresponding to the acromion at 320 °C in MCM-*g*-PGMA. Due to a ring opening reaction between the epoxy group and NH_2_ in AB15C5 to obtain AB15C5 modified cellulose microsphere, the shoulder peak at 320 °C disappeared in MCM-*g*-AB15C5, indicating the introduction of crown ether. DTA thermograms for MCM-*g*-PGMA and MCM-*g*-AB15C5 show an obvious endothermic peak at 380 °C in the DTA curve. Moreover, the TGA results show the order of residual mass for different samples was MCM > MCM-*g*-AB15C5 > MCM-*g*-PGMA, it may be attributed to that hydroxyl content of the samples is proportional to their residual mass of solid carbon. For epoxy-functionalized cellulose, the breakage of PGMA from the cellulose resulted in a higher weight loss with a lower content of residual mass than cellulose [27]. 

**Figure 4:**
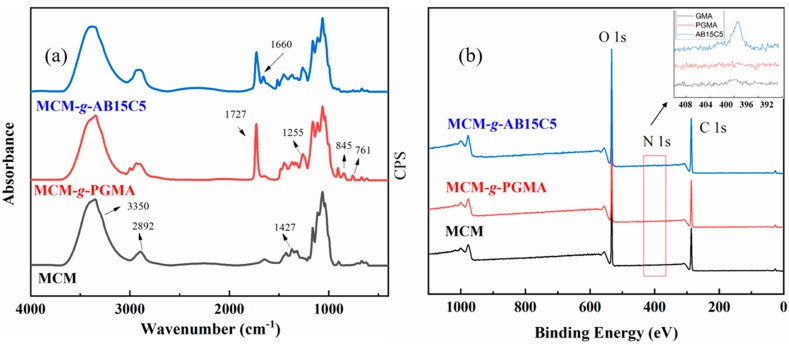
FTIR spectra (**a**) and XPS spectra (**b**) for MCM, MCM-*g*-PGMA and MCM-*g*-AB15C5.

FTIR spectroscopy was used to analyze the chemical structure of MCM, MCM-*g*-PGMA and MCM-*g*-AB15C5. As shown in Figure 4a, the broad band around 3350 cm^−1^ in MCM was attributed to the stretching vibration of abundant hydroxyl groups of cellulose, and the bending vibration of C-H existed at 2892 and 1427 cm^−1^. An obvious absorption band at 1727 cm^−1^ was observed in the FTIR spectra of MCM-*g*-PGMA, which was corresponding to the bending vibration of C=O; the bands at 761, 845 and 1255cm^−1^ were due to the epoxy groups of PGMA [28], these bands confirmed that PGMA was grafted onto MCM successfully. Compared with the FTIR spectra of MCM-*g*-PGMA, besides the band of C=O, a new band at 1660 cm^−1^ appeared in MCM-*g*-AB15C5 and it is assigned to the vibration of benzene in AB15C5, indicating the successful introduction of AB15C5 onto the modified cellulose microspheres [13,25].

In order to further determine the composition of the microspheres and the content of AB15C5 immobilized in adsorbent, MCM, MCM-*g*-PGMA and MCM-*g*-AB15C5 were analyzed by X-ray photoelectron spectroscopy (XPS) and elemental analysis. As can be seen from the XPS pattern (Figure 4b), MCM mainly contains C and O elements. After the grafting of PGMA, no obvious new peak could be found. The N1s peak appeared in the MCM after immobilizing AB15C5, also indicating that the successful synthesis of the MCM-*g*-AB15C5 microspheres. At the same time, the elemental analysis showed that the nitrogen content of MCM-*g*-AB15C5 was 1.4% (1 mmol·g^−1^). 

**Figure 5:**
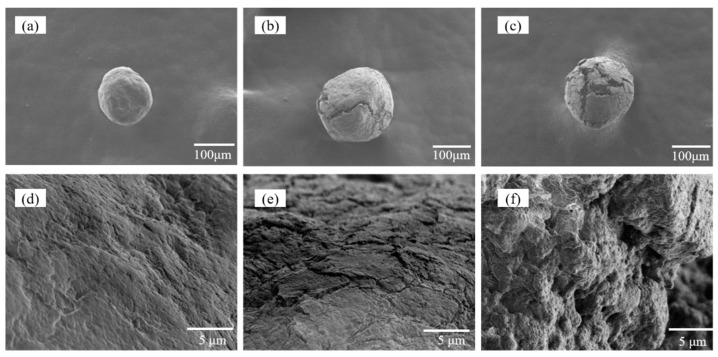
SEM images of (**a**,**d**) MCM, (**b**,**e**) MCM-*g*-PGMA, (**c**,**f**) MCM-*g*-AB15C5.

Since the morphology of microspheres may affect the adsorption property, the morphology of original MCM, MCM-*g*-PGMA and MCM-*g*-AB15C5 was investigated by SEM. As shown in Figure 5, compared with the original MCM (Figure 5a,d), the surface of grafted MCM-*g*-PGMA (Figure 5b,e) was more irregular and rough, and the average diameter of MCM-*g*-PGMA increased from 180 to 200 μm, and after the immobilizing AB15C5, some cracks and holes appeared in the MCM-*g*-AB15C5 (Figure 5c,f). Under studied conditions, the morphology of MCM-*g*-AB15C5 still remained good spheres shape with an average particle size of 210 µm (Figure 5c).

Therefore, all the above characterizations indicate that the PGMA was grafted onto MCM and finally the AB15C5 has been successfully immobilized into the MCM. Then the MCM-*g*-AB15C5 with the amount of AB15C5 (1.0 mmol·g^−1^) was used for the following study on the adsorption performance towards Li^+^ and its isotopes.

### 2.2. Adsorption Uptake of Li^+^ And Separation Factor of ^6^Li/^7^Li

**Figure 6:**
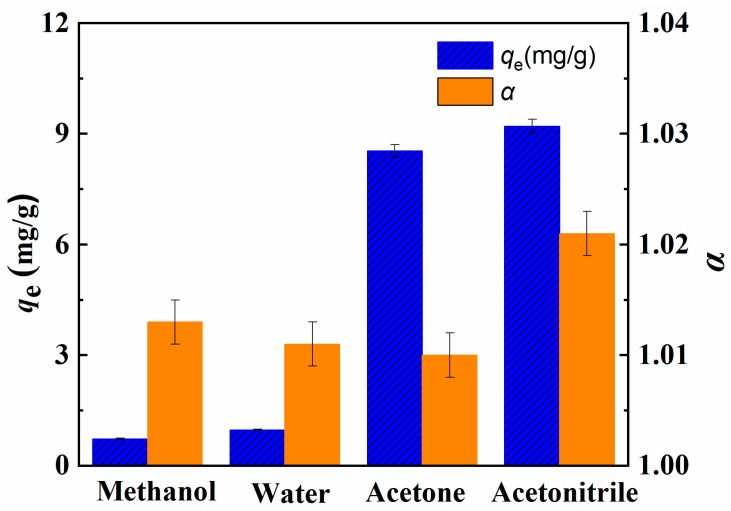
Effect of solvent on the adsorption uptake of Li^+^ and separation factor of isotopes by MCM-*g*-AB15C5 (adsorption condition: CF_3_COOLi, [Li^+^] = 1 g·L^−1^, 25 °C).

It is well known that crown ethers show specific ability to form stable complexes with alkali metals. The selectivity of crown ethers for metal ions is related with the number of oxygen atoms in the ring of crown ether. It was reported that B15C5 could form a complex with the Li^+^ with relatively high stability constant [29,30,31]. The separation of Li^+^ and its isotopes using MCM-*g*-AB15C5 were studied by batch adsorption method. According to previous reports, the stability of the complexes between macrocyclic ligand and Li^+^ is affected by solvents with different polarities [32,33]. So we study the effect of solvent on the adsorption performance of resultant MCM-*g*-AB15C5 firstly. The results were shown in Figure 6. Different solvents besides water, such as acetonitrile, methanol and acetone were used as solvent of adsorption solution. In comparison, the MCM-*g*-AB15C5 adsorbents exhibited the strongest adsorption affinity to Li^+^ in acetonitrile, Li^+^ uptake increased in the order acetonitrile > acetone > water > methanol, and the separation factor (α) of ^6^Li/^7^Li increased in the order acetonitrile > methanol > water > acetone. In acetonitrile, MCM-*g*-AB15C5 has the highest adsorption uptake of Li^+^ (10.88 mg·g^−1^) and the highest separation factor was 1.022 ± 0.002 of ^6^Li/^7^Li. The effect of solvent on the adsorption ability of MCM-*g*-AB15C5 could be explained by the enthalpic value stabilized with the solvent polarity [34], due to that less energy was consumed in the cation desolvation process in solvents with lower dielectric constants. For the adsorption of Li^+^ onto MCM-*g*-AB15C5, the solvent with a low dielectric constant is a favor to remove the water effect on the interaction between metal ions and AB15C5 immobilized in MCM-*g*-AB15C5. However, the effect of solvent on the separation of lithium isotopes seems more complicated, and the result could not be explained by the difference of polarity of solvent only, it was worthy to be studied in the future.

**Figure 7:**
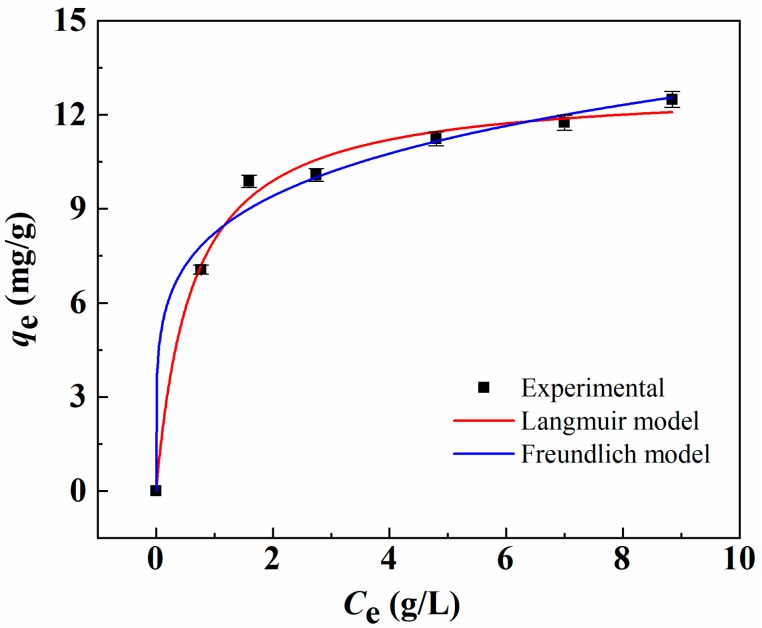
The adsorption isotherm curves for Li^+^ onto MCM-*g*-AB15C in acetonitrile fitting with different models.

Furthermore, the concentration to reach maximum adsorption was of importance to describe the interaction performance between Li^+^ and MCM-*g*-AB15C5. Figure 7 shows the adsorption behavior of MCM-*g*-AB15C5 microspheres with different initial Li^+^ concentrations in the acetonitrile. Obviously, the Li^+^ uptake increased rapidly with the increasing of Li^+^ concentration, and gradually approached to equilibrium due to complete occupation of the accessible AB15C5 ligands on the surfaces of MCM-*g*-AB15C5 adsorbents. In order to study the relationship between the equilibrium adsorption amount and the equilibrium concentration of Li^+^, the following adsorption isotherm experiments were carried out. The adsorption isotherm curve of MCM-*g*-AB15C5 towards Li^+^ at room temperature is shown in Figure 7. The Langmuir isotherm adsorption model is shown in Equation (1):

(1) $$q_{e}=\frac{q_{m}K_{L}C_{e}}{1+K_{L}C_{e}}$$

where *q*_m_ is maximum adsorption capacity. *C*_e_ and *K*_L_ are the equilibrium concentration of solution Li^+^ and the Langmuir adsorption constant [22], respectively. The Freundlich isotherm adsorption model can be shown in Equation (2):

(2) $$q_{e}{\;=\;K}_{F}C_{e}^{1/n}$$

where *K*_F_ and *1/n* represent the Freundlich adsorption coefficient, and the complexity of adsorption process, respectively.

The fitting results of the Langmuir model and Freundlich model for the adsorption of Li^+^ onto MCM-*g*-AB15C5 are listed in Table 1. The experimental data was fitted better with Langmuir model (*R*^2^ = 0.993) than Freundlich model (*R*^2^ = 0.987). Considering that the MCM-*g*-AB15C5 is a heterogeneous material, which is suitable for Freundlich model, we thought that the adsorption behavior of the adsorbent could be described with both Langmuir and Freundlich isotherm models [35].

Comparison between our works with the reported literature for the adsorption of Li^+^ by using crown ether is presented in Table 2. It shows that MCM-*g*-AB15C5 has much higher adsorption capacity than the reported similar adsorbents. 

Since the content of AB15C5 immobilized in MCM-*g*-AB15C5 adsorbent used in the batch adsorption was 1 mmol·g^−1^, the calculated highest adsorption uptake should be less than 7 mg·g^−1^ for Li^+^ assuming the formation of 1:1 complex. Whereas, the adsorption capacity obtained by Langmuir model was up to 12.9 mg·g^−1^, which was almost twice as much as the calculated maximum adsorption uptake. After analyzing the chemical structure of resultant MCM-*g*-AB15C5, we proposed that besides that the ring of crown ether provides a complexing interaction with Li^+^, the pseudo-heterocyclic structure of side groups in the AB15C5 perhaps formed during the adsorption in acetonitrile due to the self-assembly of crown ethers, which could complex with Li^+^ as well [36]. 

**Figure 8:**
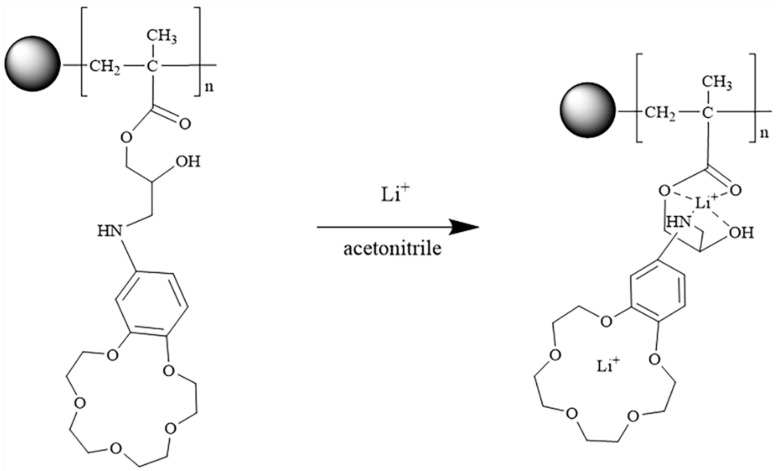
Possible adsorption mechanism of MCM-*g*-AB15C5 towards Li^+^.

Thus, the complexation ratio of resultant adsorbents and Li^+^ up to 1:2 was achieved under suitable conditions. Figure 8 shows the possible complexation interaction between adsorbents and Li^+^ during the adsorption in acetonitrile. Therefore, in this case, novel functionalized cellulose microspheres with double ligand groups towards Li^+^ fabricated by a simple method could remove the Li^+^ efficiently from acetonitrile solutions.

**Figure 9:**
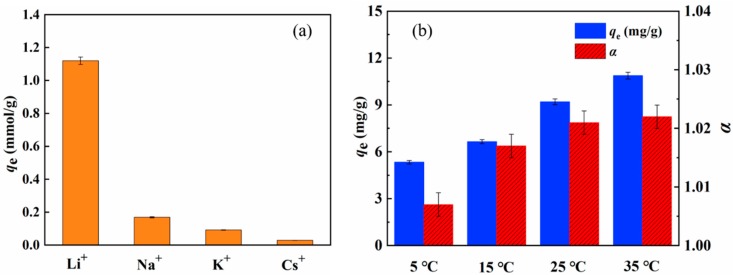
Selective adsorption (**a**) and effect of adsorption temperature (**b**) on the adsorption of Li^+^ and separation factor of isotopes by MCM-*g*-AB15C5.

Additionally, the selectivity of MCM-*g*-AB15C5 to Li^+^ was evaluated, which is important for their practical application. Different alkali metal ions, including Na^+^, K^+^ and Cs^+^ with a concentration of 1.0 g·L^−1^ for each ion were introduced into the acetonitrile as coexisting ions. As shown in Figure 9a, the MCM-*g*-AB15C5 absorbent exhibits selective binding ability towards Li^+^ against the competing metal ions with the order Li^+^ >> Na^+^ > K^+^ > Cs^+^. It is attributed to the cavity size fitting of the AB15C5 ligands with Li^+^. The selectivity factors (SF) were calculated to be SF_Li/Na_ = 2.4, SF_Li/K_ = 2.8, and SF_Li/Cs_ = 3.1, respectively. The SF can be described by the following Equation (3) [20].

(3) $$\mathrm{SF}=\frac{q(Li)\times c(M)}{q(M)\times c(Li)}$$

where *q*(Li) and *q*(M) are the equilibrium capacity of Li^+^ and the other metal ion, respectively. And *c*(M) and *c*(Li) (mmol·g^−1^) represent to the equilibrium concentration of the corresponding metal ions and Li ions, respectively.

In order to deeply understand the adsorption behavior of resultant MCM-*g*-AB15C5 towards Li^+^ in acetonitrile, the influence of adsorption temperature on the adsorption ability of the MCM-*g*-AB15C5 was measured in the range of 5 to 35 °C. Figure 9b shows that increasing adsorption temperature could enhance the adsorption capacity of Li^+^ and separation factor of ^6^Li/^7^Li simultaneously, indicating that the adsorption and separation of lithium isotopes by MCM-*g*-AB15C5 in acetonitrile was an endothermic process.

**Figure 10:**
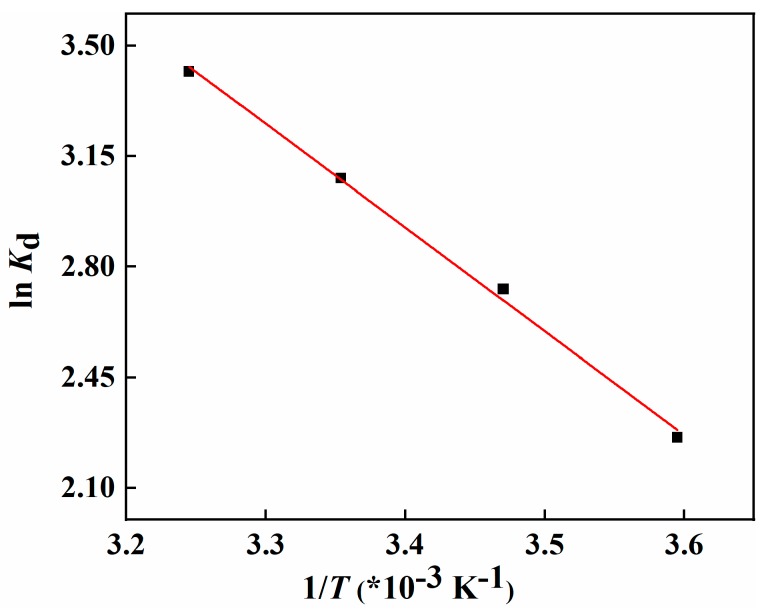
Fitted curves of ln *K*_d_ and 1/T (* 10^–3^·K^−1^) by using the Van’t Hoff equation.

The thermodynamics of the adsorption process of MCM-*g*-AB15C5 towards Li^+^ and its isotopes was investigated. Figure 10 shows the adsorption uptake of Li^+^ onto MCM-*g*-AB15C5 increases with temperature from 5 to 35 °C. Based on the proposed adsorption mechanism (Figure 8), with the increase of adsorption temperature, the mobility of grafting macromolecular in the MCM-*g*-AB15C5 should be improved to form a suitable self-assembly conformation to complex with Li^+^. In addition, the increase of adsorption temperature will accelerate the diffusion of Li^+^ into the microspheres to contact with ligands of adsorbent inside. The equilibrium constant was calculated by Equation (4) for Van’t Hoff Equation (5) and Gibbs function Equation (6) that were used to calculate the thermodynamic parameters.

(4) $$K_{d}=\frac{q_{e}}{c_{e}}$$

(5) $$\ln K_{d}=\frac{-\Delta H^{}}{RT}+\frac{\Delta S^{}}{R}$$

(6) $$\Delta G^{}=\Delta H^{}-T\Delta S^{}$$

where *K*_d_ is the thermodynamic equilbrium constant calculated by Equation (4). *R* is the universal constant of gas, which is 8.314 J·mol^−1^·K^−1^.

According to the calculated results, the value of ΔH and ΔS were 27.30 kJ·mol^−1^ and 117.13 J·mol^−1^·K^−1^, respectively, indicating that the adsorption process by MCM-*g*-AB15C5 toward Li^+^ is an endothermic process driven by entropy gain. At the temperature range of 278.15–308.15 K, the value of ΔG was negative in the range of −5.28 to −8.79 kJ·mol^−1^ indicating the spontaneous adsorption reaction happened, when the temperature increased from 278.15 to 308.15 K. Previous research reported that the value of ΔH between 20.9 to 418.4 kJ·mol^−1^ corresponds to a chemisorption process [39]. Thus, the adsorption process of as-synthesized MCM-*g*-AB15C5 toward Li^+^ was a chemisorption process.

## 3. Materials and Methods 

### 3.1. Materials

4′-aminobenzo-15-crown-5 (AB15C5, 98%) and the microcrystalline cellulose microspheres (MCM) with an average diameter 180 μm were purchased from Beite Tec. Co. and Lisui Chemical Co, respectively. Glycidyl methacrylate (GMA, 99%) was obtained from J&K Co. All reagents were analytical-grade chemicals and used without further purification.

### 3.2. Preparation of MCM-g-PGMA and MCM-g-AB15C5

Firstly, MCM was exposed to γ-ray radiation from ^60^Co source (Peking University, Beijing, China) at dose of 19.8 kGy with a dose rate of 330 Gy·min^−1^. And then, the irradiated MCM was put in 5% tween-20 aqueous solution containing GMA, and stirred in the nitrogen atmosphere for 10 min. Afterward, the emulsion grafting polymerization was carried out in a shaking incubator (with a frequency of 120 rpm) at 50 °C for 2 h. The resultant solid product was collected by filtration and washed alternately by DMF, ultrapure water and acetone at least three times, respectively. Finally, the prepared products were vacuum dried at 45 °C for 24 h. Degree of grafting (DG) was employed to evaluate the amount of GMA grafted onto MCM, and calculated by Equation (7) as follows:

(7) $$\mathrm{DG}\;\left(\%\right)=\frac{W_{g}-W_{0}}{W_{0}}\times 100$$

where W_0_ and Wg are the dry weights of the original MCM and MCM-*g*-PGMA, respectively. Consequently, 0.33 g MCM-*g*-PGMA was dispersed in 25 mL of DMF solution containing AB15C5 in shaker at 80 °C for 48 h. The MCM-g-AB15C5 was obtained after washing products by DMF, ultrapure water and acetone, respectively, and followed by a vacuum drying at 50 °C for 24 h.

### 3.3. Characterization

Since the MCM and MCM-*g*-PGMA don’t contain nitrogen element, the content of AB15C5 groups in MCM-*g*-AB15C5 could be characterized by measurement of nitrogen content using Vario Elemental Analyzer (Elementar Analysensysteme GmbH, Langenselbold, Germany), and thereby the standard deviation of nitrogen content was estimated to be 5%. Fourier Transform Infrared (FTIR, Spotlight 200, PerkinElmer, Waltham, MA, USA) spectra was used to identify the chemical group of samples in the range of 4000–400 cm^−1^. Thermo Gravimetric Analyzer (TGA, Q600 SDT, TA Instruments, New Castle, DE, USA) was used to study the thermal stabilities of MCM, MCM-*g*-PGMA, MCM-*g*-AB15C5 that were heated from 20 to 600 °C at a heating rate of 20 °C/min under nitrogen atmosphere. The surface morphology of the MCM, MCM-*g*-PGMA and MCM-*g*-AB15C5 samples was observed by Scanning Electron Microscope (SEM, S-4800, Hitachi, Tokyo, Japan). X-ray Photoelectron Spectroscopy (XPS) measurements were performed on Axis Supra (Kratos Analytical, Manchester, UK).

### 3.4. Adsorption of Li^+^ and Its Isotopes

Adsorption of Li^+^ was performed by a batch operation. Typically, 50 mg MCM-*g*-AB15C5 was mixed with 1.0 mL 1.0 g·L^−1^ CF_3_COOLi solution and stirred in a shaking incubator for 48 h at 25 °C. The concentration of residual Li^+^ in solution was measured by ICP-AES (Prodigy, Leeman, Hudson, NH, USA). In the work, the ICP-AES was used to measure the concentration of Li^+^, and thereby the standard deviation of Li^+^ concentration was estimated to be 2%. Adsorption uptake of Li^+^ (*q*_e_) was calculated by the following Equation (8):

(8) $$q_{e}{=(C}_{0}{-C}_{e})\times V/m$$

where *C*_0_ (mg·L^−1^) and *C*_e_ (mg·L^−1^) are the initial and residual concentration of Li^+^ in solvent phases, respectively. *V* (mL) is the volume of the solution, and *m* (mg) is the mass of MCM-*g*-AB15C5.

In order to determine the separation factor of lithium isotopes, the resultant MCM-*g*-AB15C5 was treated in nitric acid, and the digested solution is diluted with ultrapure water to reduce the concentration of Li^+^ to ppb level, and a high resolution ICP-MS (Element XR, Thermo Scientific, Waltham, MA, USA) was used to measure the lithium isotope ratio in the samples, at least one hundred data were collected during the measurement to give an average value of isotope ratio, moreover, each sample was repeated three times to give an average value and the standard deviation of separation factor is 0.2%. Separation factor (α) of lithium isotopes was defined as follows Equation (9):

(9) α=(L6i/L7i)s(L6i/L7i)w

where (^6^Li/^7^Li) represents the isotopic ratio. The subscripts ‘s’ and ‘w’ represent the digestion solution of the solid phase and the solvent phase, respectively.

## 4. Conclusions

This study reported a new kind of 4′-aminobenzo-15-crown-5 functionalized cellulose microsphere (MCM-*g*-AB15C5) adsorbents for efficient separation of Li^+^ and ^6^Li/^7^Li. This MCM-*g*-AB15C5 adsorbent was prepared successfully via a pre-irradiation-induced emulsion grafting and followed by chemical functionalization method with GMA as monomer and AB15C5 as a ligand. Due to the specific structure of MCM-*g*-AB15C5, the adsorption capacity of Li^+^ onto MCM-*g*-AB15C5 was 12.9 mg·g^−1^ determined by Langmuir model. The excellent adsorption capacity of MCM-*g*-AB15C5 is attributed to the ring of crown ether and the pseudo-heterocyclic structure of side groups in the AB15C5 perhaps formed during the adsorption in acetonitrile, leading to the complexation ratio of adsorbents and Li^+^ up to 1:2. The separation factor for ^6^Li/^7^Li at room temperature was 1.022 ± 0.002, which is comparable with the result reported in recent literatures. The solvent with low polarity is favorable for the adsorption of Li^+^, and the MCM-*g*-AB15C5 adsorbents have good selectivity towards Li^+^ in the presence of Na^+^, K^+^, and Cs^+^, the adsorption and separation of lithium isotopes by AB15C5 functionalized cellulose microspheres in acetonitrile was an endothermic and chemisorption process (ΔH = 27.30 kJ·mol^−1^). The prepared MCM-*g*-AB15C5 adsorbents are promising adsorbents for the adsorption of Li^+^ and its isotope separation.

## Figures and Tables

**Table 1:** The fitting parameters by the Langmuir model and Freundlich model for Li^+^ onto MCM-*g*-AB15C5.

Langmuir			Freundlich		
*K*_L_ (L·mg^−1^)	*q*_m_ (mg·g^−1^)	*R* ^2^	*K*_f_ ((mg·g^−1^) (L·mg)^1/*n*^)	*n*	*R* ^2^
1.63	12.90	0.993	8.22	5.15	0.987

**Table 2:** Comparison of adsorption capacity of MCM-*g*-AB15C5 with the published adsorbents for Li^+^.

Adsorbents	Adsorption Capacity (mg·g^−1^)	Reference
Macroporous polymer foam (2-methylol-12-crown-4)	3.15	Huang et al. (2018) [37]
Imprinted hierarchical porous silica (12-crwon-4)	0.166	Xu et al. (2018) [38]
Glass fiber mats (4′-aminobenzo-15-crown-5)	6.46	Wang et al. (2018) [31]
Mesoporous silica/polymer hybrids (4′-aminobenzo-15-crown-5)	6.97	Liu et al. (2017) [12]
Mesoporous silicas (4′-aminobenzo-15-crown-5)	5.14	Liu et al. (2016) [11]
Multi-walled carbon nanotubes (Hydroxy-dibenzo-14-crown-4 ether)	2.11	Torrejos et al. (2015) [10]
MCM-*g*-AB15C5	12.90	This work
